# Tumour treating fields therapy for glioblastoma: current advances and future directions

**DOI:** 10.1038/s41416-020-01136-5

**Published:** 2020-11-04

**Authors:** Ola Rominiyi, Aurelie Vanderlinden, Susan Jane Clenton, Caroline Bridgewater, Yahia Al-Tamimi, Spencer James Collis

**Affiliations:** 1 Weston Park Cancer Centre, Department of Oncology & Metabolism, The University of Sheffield Medical School, Sheffield, UK; 2 Department of Neurosurgery, Royal Hallamshire Hospital, Sheffield Teaching Hospitals NHS Foundation Trust, Sheffield, UK; 3 Department of Clinical Oncology, Weston Park Hospital, Sheffield Teaching Hospitals NHS Foundation Trust, Sheffield, UK

**Keywords:** Chemotherapy, Physics, CNS cancer, Targeted therapies, Radiotherapy

## Abstract

Glioblastoma multiforme (GBM) is the most common primary brain tumour in adults and continues to portend poor survival, despite multimodal treatment using surgery and chemoradiotherapy. The addition of tumour-treating fields (TTFields)—an approach in which alternating electrical fields exert biophysical force on charged and polarisable molecules known as dipoles—to standard therapy, has been shown to extend survival for patients with newly diagnosed GBM, recurrent GBM and mesothelioma, leading to the clinical approval of this approach by the FDA. TTFields represent a non-invasive anticancer modality consisting of low-intensity (1–3 V/cm), intermediate-frequency (100–300 kHz), alternating electric fields delivered via cutaneous transducer arrays configured to provide optimal tumour-site coverage. Although TTFields were initially demonstrated to inhibit cancer cell proliferation by interfering with mitotic apparatus, it is becoming increasingly clear that TTFields show a broad mechanism of action by disrupting a multitude of biological processes, including DNA repair, cell permeability and immunological responses, to elicit therapeutic effects. This review describes advances in our current understanding of the mechanisms by which TTFields mediate anticancer effects. Additionally, we summarise the landscape of TTFields clinical trials across various cancers and consider how emerging preclinical data might inform future clinical applications for TTFields.

## Background

Glioblastoma (GBM) is the most common and aggressive type of primary brain tumour, causing roughly 2500 deaths each year in the United Kingdom^1^ and the majority of ~200,000 deaths related to tumours of the central nervous system worldwide each year.^2,3^ The current standard of care for patients with GBM consists of maximal surgical resection followed by radiotherapy and chemotherapy with temozolomide (TMZ). However, even with this combination of treatment, the median overall survival (OS) is around 10–16 months, with fewer than 10% of patients surviving for 5 years or more from the time of diagnosis,^4,5^ and this scenario has improved very little over the past four decades.^6^ There is, therefore, a critical need for more effective treatment strategies to improve outcomes for patients faced with this devastating diagnosis.

Tumour-treating fields (TTFields) represent an emerging non-invasive anticancer therapeutic modality that involves the transcutaneous delivery of low-intensity (1–3 V/cm), intermediate-frequency (100–300 kHz), alternating electric fields (the approach is also known as alternating electric field therapy) that exert biophysical force on charged and polarisable molecules known as dipoles. The beneficial effects of TTFields therapy are influenced by treatment duration (with evidence that application >18 h/day improves survival^7^), electrical field intensity (where increased intensity confers greater reduction in cell proliferation) and electrical field frequency,^8^ which varies between cancer types—in the case of glioma cells, TTFields are clinically delivered at an optimum frequency of 200 kHz.^9^

**Fig. 1:**
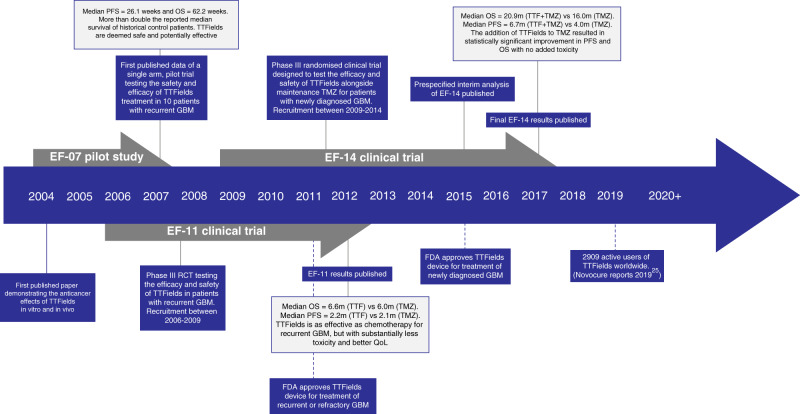
Historical timeline of the emergence of TTFields as novel therapy for GBM patients. In 2004, the first paper demonstrating the anticancer effects of TTFields in vitro and in vivo was published.^9^ Following these promising preclinical data, a number of clinical trials investigating the safety and efficacy of TTFields for the treatment of GBM were completed (details described at each relevant date), leading to the approval in 2011 and 2015 of TTFields for the treatment of recurrent and newly diagnosed GBM, respectively.

The frequency of the alternating field has also been shown to provide different biological effects. Low-frequency electric fields (<1 kHz) influence cell membrane polarisation and can alter the behaviour of excitable tissue, such as action potential firing in neuronal cells.^10^ On the other hand, high-frequency fields (>500 kHz) cause charged and/or polar molecules inside cells to vibrate, creating friction and causing kinetic energy to transfer between molecules, which can be radiated out as thermal energy, leading to tissue heating.^11^ Intermediate-frequency alternating electric fields (100–500 kHz) do not generate enough thermal energy to cause significant tissue heating and alternate too quickly to trigger action potential firing, and were consequently originally thought to lack any beneficial effects.^12^ However, Kirson et al. demonstrated that low-intensity alternating electric fields delivered at 100–300 kHz successfully inhibited cancer cell growth, both in vitro (using cell lines derived from melanoma, glioma, lung, prostate and breast cancer) and in vivo, by interfering with microtubule polymerisation during mitosis.^9^ These findings led to the first pilot study (EF-07) in GBM patients launched in 2004^13^ and, eventually, to the development of TTFields as a strategy for treating cancer (Fig. 1).

It has subsequently emerged that, in addition to its antimitotic effects, TTFields can influence a spectrum of biological processes, including autophagy, DNA repair, antitumour immunity and tumour cell migration, in addition to altering cell membrane, and potentially blood–brain barrier (BBB) permeability. This review examines the emergence of TTFields as a therapeutic modality to treat GBM and highlights molecular mechanisms that are likely to contribute to its anticancer efficacy. We also summarise the current landscape of TTFields clinical trials across various cancer types and consider how emerging preclinical data might inform future applications for TTFields in the clinic.

## TTFields as an emerging therapeutic modality

The EF-14 trial represents a landmark study, as it was the first trial in a decade to show an increase in OS for patients with newly diagnosed GBM since the addition of temozolomide (TMZ) chemotherapy to standard care.^14–17^ Following randomisation at the end of chemoradiotherapy, the addition of TTFields to maintenance of TMZ chemotherapy significantly increased median OS by 4.9 months (20.9 vs 16.0 months with TMZ alone).^16,17^ Importantly, the addition of TTFields was not associated with any significant increase in rates of systemic adverse events (48% vs 44% with TMZ alone, *P* = 0.58), and the continuous usage of TTFields appears to be associated with maintained or enhanced quality of life.^18–20^ Data from the EF-14 trial led to the approval of TTFields by the FDA in 2015 for newly diagnosed GBM.^21^

### TTFields delivery

**Fig. 2:**
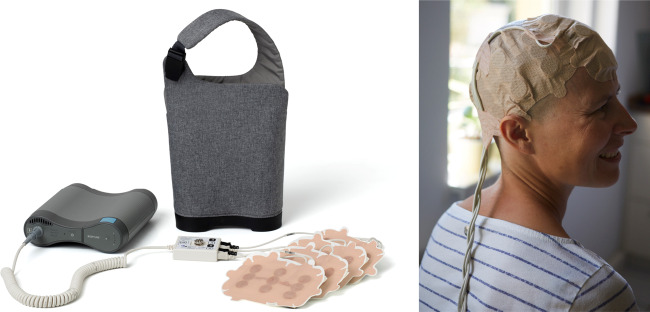
The Optune system. Left: the Optune TTFields delivery system consists of four transducer arrays, a field generator and a power source. Right: a patient wearing the Optune system. Images taken from Novocure, 2020.^36^

The most widely used clinical TTFields delivery system, Optune (Novocure™), consists of four transducer arrays, a field generator and a power source (shown in Fig. 2). For GBM, the four transducer arrays are attached in pairs, orthogonally to the patient’s scalp. The patient’s head must be shaved consistently to allow optimal contact of the transducer arrays with the scalp, and optimal array positioning is determined using NovoTAL™ (Novocure Ltd., Haifa, Israel) simulation software based on the location of the tumour and the size and shape of the patient’s head.^22^ Each transducer array is made up of nine ceramic discs, each with a superficial hydrogel coating to improve conductivity with the skin. The field generator delivers alternating electric fields through the transducer arrays across the brain and to the tumour site.

The main adverse event of TTFields is irritant or allergic contact dermatitis at the site of transducer array attachment resulting from prolonged exposure to sweat, hydrogel, adhesive or a combination of these factors. However, skin complications are usually of low grade (grade 1–2 adverse events) and can easily be managed by topical corticosteroids, modification of array positioning and/or protecting the skin with sterile dressing pads.^23^

### The cost-effectiveness of TTFields

Important financial considerations are associated with incorporating TTFields therapy into the standard of care for GBM patients. Presently, Novocure, the sole producer of the therapeutic TTFields delivery systems, rents Optune to patients for a total monthly cost of around $21,000 (subject to discounts negotiated by healthcare providers/payers).^24^ This cost covers the TTFields delivery system, and includes transducer arrays, array layout planning, patient/physician training and 24-h technical support.^25^ Additional expenses associated with implementing TTFields might include additional staff and training,^22^ and costs associated with managing treatment-related morbidities.^26^

There have been three major studies estimating the costs associated with adding TTFields to the standard-of-care therapy for GBM, all of which use EF-14 trial data. Bernard-Arnoux et al.^27^ used interim EF-14 data, while Connock et al.^28^ and Guzauskas et al.^29^ used the trial’s final results. During economical modelling, the assumptions made by Bernard-Arnoux et al. and Connock et al. were based on a French National Health Insurance perspective, while analyses by Guzauskas et al. were based on the US healthcare perspective. All three studies relied on the full list price of TTFields therapy and therefore do not incorporate potential discounts negotiated by healthcare payers.

Bernard-Arnoux et al. estimated 0.34 life years gained (LYG) from the addition of TTFields to maintenance of TMZ, with an added cost of €185,476, while Connock et al. estimated 0.604 LYG with an added cost of €453,848. These two studies then estimated the incremental cost-effectiveness ratio (ICER, a summary measure that compares the economic value of a particular intervention with another expressed as cost per LYG) to be €549,909 and €510,273, respectively. Both studies analysed survival using statistical models that were unable to account for changing (decreasing) hazard rates as patients live longer. This is an important limitation since epidemiological data suggest that as a patient survives longer, the ongoing probability of death reduces. For example, analysis of the US SEER database demonstrated patients alive 5 years after diagnosis had a 70.4% probability of surviving to 10 years post diagnosis.^30^ Therefore, although data from the EF-14 trial suggest that addition of TTFields may increase 5-year OS from 5% to 13%, the studies by Bernard-Arnoux et al. and Connock et al. did not fully account for the impact of long-term survivors beyond the trial period. This resulted in reported incremental lifetime survival benefits (the LYG) close to the median OS benefit observed within the trial period. By contrast, Guzauskas et al. integrated EF-14 data with external GBM epidemiology data and US life expectancy data to estimate long-term conditional survival (similar integration of oncology trial and epidemiological data to model long-term survival has previously been considered by NICE in its decision to licence ipilimumab for metastatic melanoma^31,32^). Consequently, the Guzauskas model estimates 1.25 LYG from adding TTFields to TMZ and estimates a corresponding ICER of $150,452.

As such, Japan, Israel and Sweden have included TTFields within their national reimbursement systems following cost-effectiveness evaluations, whilst Germany has approved TTFields for national reimbursement based on a clinical comparative effectiveness review without respect to costs. As noted above, the method of estimating future survival beyond the time observed in the trial is a critical assumption within any model. Healthcare payers that prefer the extrapolated constant hazard rate models of Bernard-Arnoux and Connock might not be willing to adopt the therapy. Adoption by healthcare systems that include considerations of cost-effectiveness as a major driver of decision-making, such as the NHS in the United Kingdom or the Australian and Canadian systems,^33,34^ is likely to depend on how those systems choose to model long-term survival.

### Clinical availability of TTFields

Although the number of patients receiving TTFields has increased since this approach was first approved for use in GBM patients (2909 patients worldwide in 2019 compared with 605 patients in 2015),^35,36^ it is thought that many more patients with approved indications could benefit from TTFields treatment (on average, 30% of eligible GBM patients currently receive TTFields in countries where the therapy is available).^29,37^ Substantial geographic variation in TTFields availability exists in the clinical usage of Optune, with the majority of patients who receive TTFields residing in the United States (roughly twice as many patients receive TTFields in the United States compared with the rest of the world).^37^ As highlighted above, high treatment costs and differences in long-term survival modelling represent major drivers of geographical variation in the usage of TTFields worldwide. Notably, some reluctance to adopt TTFields within the neuro-oncology community also exists; this might be fuelled by a range of factors. Firstly, the high cost of TTFields therapy (discussed above) may represent a barrier to adoption at an individual or national level. Secondly, valid concerns have been raised that patients in the control group of the EF-14 trial did not receive any placebo treatment,^16^ such as via a sham TTFields device. However, requiring patients to wear a sham device (ideally > 18 h per day) with no potential for benefit would likely present its own ethical challenges,^38^ and objective endpoints such as OS (which demonstrated survival benefit with TTFields in the EF-14 trial) are unlikely to be influenced substantially by the lack of placebo or blinding. Thirdly, a perceived burden of patients having to carry and wear the device with high compliance may contribute to reluctance to adopt or prescribe TTFields; nevertheless, objective data suggest that quality of life in these patients is not reduced.^19,20^ Critically, much reluctance to adopt TTFields may stem from the fact that the mechanisms of action for TTFields are currently less well-defined relative to more established therapeutic modalities.^39^ It can be expected that, as technologies continue to evolve and as competing products enter the market, TTFields might become increasingly affordable. Additionally, any enhancement of the therapeutic efficacy of TTFields might also improve the ICER and facilitate TTFields uptake by healthcare systems that currently do not deem the technology to be cost-effective, including the NHS (NICE^40^). To improve the efficacy of TTFields, an improved understanding of the diverse mechanistic effects of this therapy and how these effects can be exploited to increase the therapeutic index of TTFields-based regimens is required.

## Molecular mechanisms for the anticancer effects of TTFields

**Fig. 3:**
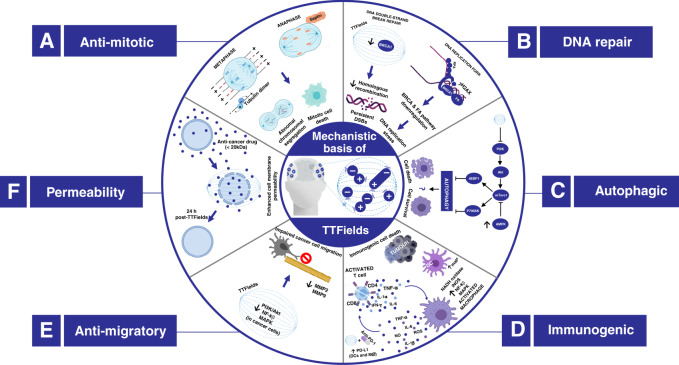
Summary of the mechanisms of action of TTFields. Low-intensity, intermediate-frequency, alternating electrical fields exert biophysical forces on a variety of charged and polarisable molecules to elicit a spectrum of biological effects. **A** Antimitotic effects: during metaphase, the electric fields are uniform, causing dipolar molecules, such as tubulin, to align with the field. TTFields therefore interfere with tubulin polymerisation and depolymerisation during metaphase. At anaphase, TTFields prevent localisation of septin proteins to the mitotic spindle and inhibit assembly of the septin complex into a ring structure at the cleavage furrow. During cytokinesis, the electric fields are non-uniform, with the fields converging on the cleavage furrow, where the field intensity is the highest. As a result, strong dielectric force is applied on polarisable objects, pushing them towards the high-intensity region. Together, these effects result in abnormal chromosome segregation and/or cell death. **B** DNA repair*.* TTFields have been shown to downregulate BRCA and Fanconi anaemia (FA) pathway genes, which have been associated with increased replication stress and increased double-strand break (DSB) formation. Additionally, homologous recombination repair (HRR) is impaired by TTFields, resulting in reduced efficiency of DSB repair. **C** Autophagy*.* TTFields have been suggested to prevent the inhibitory effects of the PI3K/Akt/mTORC1 signalling pathway on autophagy, resulting in increased activation of autophagy with TTFields therapy. Further studies are needed to ascertain whether autophagy is activated as a cell survival or cell death signal in response to TTFields. **D** Antitumour immunity*.* TTFields stimulates macrophages (Mø) to secrete reactive oxygen species (ROS), nitric oxide (NO) and proinflammatory cytokines such as interleukin (IL)-1β, tumour necrosis factor (TNF)-α and IL-6. Additionally, TTFields promote immunogenic cell death via dendritic cell (DC) recruitment and maturation (mat), ultimately leading to an increase in the accumulation of CD4 + and CD8 + T cells at the tumour site. The combination of TTFields with anti-PD-1 therapy might enhance PD-L1 expression in infiltrating DCs and macrophages to further enhance antitumour immunity. **E** Anti-migratory*.* TTFields reduce the capacity of cancer cells for migration and invasion through nuclear factor (NF)-κB-, mitogen-activated protein kinase (MAPK)- and phosphatidylinositol 3-kinase (PI3K)/Akt-dependent mechanisms. **F** Cell membrane permeability. TTFields increase cell membrane permeability by increasing the number and size of holes in the cell membrane, thereby potentially enhancing sensitivity to chemotherapeutic drugs.

Increasing evidence suggests the therapeutic effects of TTFields may be associated with a diverse range of intracellular mechanisms. This is perhaps unsurprising considering the abundance of a broad range of charged and polarisable molecules within cells upon which TTFields could exert biophysical forces. Although the spectrum of effects elicited remains incompletely understood, emerging data suggest that in addition to the antimitotic effects of TTFields, a multitude of biological processes, including DNA repair, autophagy, cell migration, permeability and immunological responses, are perturbed by TTFields to elicit anticancer effects. A summary of the reported molecular mechanisms by which TTFields impacts tumour cell toxicity is shown in Fig. 3.

### Antimitotic effects of TTFields

The principle mechanisms of action through which TTFields application is thought to mediate its therapeutic effects are antimitotic. The rapidly dividing nature of cancer cells, relative to normal tissue, underlies their specific sensitivity to TTFields. Furthermore, comparison of TTFields susceptibility between various cancer cell lines demonstrates an inverse correlation between the typical doubling time of cell lines and TTFields-induced cell death.^41^ However, the effects of TTFields are also dependent on the specific frequency of alternating electric fields applied;^9^ therefore, calibration of an optimal frequency to exert cytotoxic effects on a specific cancer cell type within the intermediate range (100–300 kHz) is also postulated to facilitate the cancer-specific effects of TTFields on mitosis. During chromosome segregation, chromosomes align at the metaphase plate, and sister chromatids are separated and pulled to opposite poles of the cell by the mitotic spindle. The mitotic spindle is formed from an array of microtubules, comprising tubulin polymers, with each tubulin subunit possessing a relatively high dipole moment.^42^ When TTFields are applied, tubulin dimers align with the electric field, which interferes with the normal microtubule polymerisation–depolymerisation process during mitosis. This, in turn, results in abnormal spindle formation, which can lead to cellular arrest in mitosis for several hours, eventually leading to mitotic cell death. In other cases, failure of the spindle assembly checkpoint (SAC),^43,44^ a mitotic checkpoint ensuring that all chromosomes are properly attached to the mitotic spindle before proceeding through to anaphase to enable correct chromosome segregation,^45^ might lead to aberrant metaphase exit, abnormal chromosome segregation, multinucleation and consequently cell death.^43,46^ Interestingly, pharmacological inhibition of the SAC key regulator monopolar spindle 1 (MPS1) kinase using the inhibitor IN-3 in combination with TTFields has been demonstrated to increase nuclear abnormalities, G2/M cell-cycle arrest and apoptotic cell death relative to either treatment used as a single therapy in glioblastoma cell lines.^47^ Furthermore, use of this combination (TTFields + IN-3) in cultured GBM cells provided a durable therapeutic response for 72 h following the cessation of TTFields, highlighting the potential clinical utility of such combinatorial strategies to resist tumour regrowth during breaks in the delivery of TTFields therapy to patients (e.g., breaks in therapy due to TTFields-associated skin toxicity).^47^ A Phase 1 clinical trial of the potent MPS1 inhibitor BAY1217389^48^ (NCT02366949) has recently been completed; therefore, future clinical studies assessing the use of SAC inhibition to enhance the effectiveness of TTFields would be feasible.

With TTFields, although the electric field is uniform in non-replicating cells, it is non-uniform in dividing cells because of the ‘hourglass’ structure adopted by dividing cells after anaphase. Non-uniform electric fields generate forces that cause dielectrophoresis, in which the field intensity is increased at the furrow during cytokinesis, causing charged and/or dipolar molecules to accumulate here.^13^ During cytokinesis, the mitotic septin complex (comprising septins 2, 6 and 7) is normally recruited to the spindle midline and cleavage furrow at anaphase, and assembles into a ring structure, where it positions the cleavage furrow to limit contraction to the equatorial plane and restricts determinants to separate cortical domains.^49,50^ The septin complex is also involved in cross-linking actin, non-muscle myosin II and RhoA, facilitating actin-based myosin contraction, which directs cleavage furrow ingression and provides the contractile forces required to physically separate the forming daughter cells from each other.^50–52^ TTFields therapy has been shown to prevent the localisation of the mitotic septin complex to the spindle midline and cleavage furrow at anaphase due to TTFields-induced dielectrophoresis. Failure to localise the septin complex appropriately also leads to abnormal chromosomal segregation, extended duration in mitosis and morphological changes in the membrane of cells that are characteristic of post-mitotic apoptotic cell death, such as cell membrane blebbing and rupture^46^ (Fig. 3A).

### Effects of TTFields on the DNA-damage response

Several studies have reported that TTFields sensitise glioma cell lines to radiotherapy. Exposure to TTFields prior to radiotherapy was shown to delay the repair of radiation-induced DNA damage, enhance mitotic catastrophe and reduce glioma cell line survival.^53,54^ Additionally, cell survival was decreased in non-small-cell lung carcinoma (NSCLC) cells treated with TTFields prior to or after radiotherapy treatment compared with either treatment alone; however, exposing cells to TTFields before radiotherapy was more toxic.^55^ These findings could have implications for the timing of TTFields application in future preclinical and clinical studies, with TTFields application prior to, or immediately after, radiotherapy likely to optimise therapeutic efficacy. TTFields therapy has also been suggested to interfere with the efficiency of DNA repair. Giladi et al. found more numerous γH2AX foci (an established marker of DNA damage) in glioma cells 24 h post radiotherapy in the combination group compared with either treatment alone. These results suggest that the increased sensitivity to radiotherapy observed with TTFields could be mediated through both an increase in DNA damage and reduced repair capacity following TTFields treatments.^53^

Consistent with these findings, differential gene expression analysis revealed that the expression of genes encoding the DNA-repair proteins BRCA1, ATRIP, MLH1, MRE11A, FANCM and FANCD2, was significantly downregulated in TTFields-treated NSCLC cell lines compared with baseline expression, and that this downregulation was more pronounced in cell lines that were more sensitive to TTFields relative to cell lines that are more resistant to TTFields.^55^ BRCA1 plays a central role in homologous recombination DNA repair (HRR), recruiting, along with BRCA2, RAD51 filaments to sites of DNA damage.^56–58^ During homologous recombination, RAD51 mediates sequence homology search and strand invasion into the sister chromatid, and prevents nucleolytic degradation of stalled replication forks.^59^ RAD51 foci can therefore be used to monitor HRR efficiency, with cells that retain RAD51 foci for 24 h following radiotherapy being associated with persistent DNA double-strand breaks (DSBs) and eventually cell death. Giladi et al. showed an increase in persistent RAD51 foci 24 h following combination treatment (radiotherapy plus TTFields) compared with either treatment alone, suggesting that the reduced repair efficiency seen with TTFields could be the result of impaired HRR following TTFields application. Notably, non-homologous end-joining repair kinetics were not affected by TTFields treatment.^53^

In addition to their role in HRR, BRCA genes cooperate with Fanconi anaemia pathway proteins to maintain DNA replication fork stability.^60^ Karanam et al. showed that replication stress was increased with TTFields, and that replication fork dynamics were impaired.^61^ Measuring the incorporation of labelled nucleotides into newly synthesised DNA strands during DNA replication serves as a robust readout for replication stress and replication fork dynamics.^62^ Karanam et al. showed that DNA fibre length was shorter in H157 and H1299 cells treated with TTFields compared with DNA fibre length in untreated cells, and the difference in DNA fibre length between groups increased over time, indicating that TTFields interfere with replication fork progression and induce replication stress. In addition, the authors demonstrated the presence of other replication-stress markers following TTFields treatments,^61^ such as increased replication protein A (RPA) foci (RPA is recruited to single-stranded DNA (ssDNA) intermediates during DNA replication, where it protects exposed ssDNA from nucleases and prevents ssDNA from reannealing^63^) and increased R-loop formation (regions of 3-stranded nucleic acid that form when a replication fork collides with the transcription machinery; these are produced at a higher rate during replication stress).^64^ Finally, the authors demonstrated reduced expression of the mini-chromosome maintenance (MCM) complex genes MCM6 and MCM10^61^ (the MCM complex functions as a DNA helicase that is crucial for replication initiation and replication fork assembly). Collectively, these data suggest that downregulation of BRCA/Fanconi anaemia pathway genes by TTFields results in an increase in replication-stress-induced DSBs and reduced DSB-repair efficiency due to impaired HRR kinetics (Fig. 3B). As cancerous cells often demonstrate overreliance on a reduced repertoire of DNA- damage response processes,^65,66^ future combinatorial strategies that exploit these TTFields-induced vulnerabilities might be particularly effective.

### Effects of TTFields on autophagy

The roles of autophagy in cancer are diverse. During the early phases of cancer initiation, the upregulation of autophagy exhibits tumour-suppressive functions, whereas autophagy can be activated to promote cancer cell survival and treatment resistance during the later stages of cancer development.^67^ Previous studies have demonstrated that TTFields-treated cells display features that are characteristic of autophagy, such as increased cell volume and granularity and the formation of double-membraned autophagosomes.^46,68–70^ When cells undergo autophagy, microtubule-associated protein light-chain 3 (LC3-I) is converted into LC3-II through lipidation by autophagy-related protein 7 (ATG7), enabling its recruitment to the autophagic vesicle membrane, where it activates ATG5, a key component in autophagic vesicle formation.^71^ As such, LC3 is often used as a marker for monitoring autophagy.^72^ Shteingauz et al. observed an increase in the LC3-II protein in cancer cells following the application of TTFields.^68^ However, increased levels of LC3-II do not always correlate with increased autophagy, and can also signify the reduced turnover of autophagosomes owing to defects in autophagosome transport and fusion of the autophagosome with the lysosome.^73^ Consequently, autophagic flux, which describes the entire process of autophagy (autophagosome formation, maturation, fusion with lysosomes and lysosomal degradation of cytoplasmic constituents) must be measured to determine the degree of autophagy. Measuring the difference in LC3-II levels in the presence and absence of a lysosome inhibitor, such as chloroquine (which inhibits autophagosome–lysosome fusion), allows the determination of how much LC3-II is degraded in a lysosome-dependent manner because it stops autophagic flux before lysosomal degradation can take place, and therefore indicates the extent of degradation that would have taken place during the treatment, reflecting the degree of autophagy.^74^ Combining chloroquine with TTFields was shown to significantly increase LC3-II levels in cells relative to control and relative to TTFields-treated cells in the absence of chloroquine, indicating that TTFields increase autophagic flux and activate autophagy.^68^

TTFields therapy has also been shown to induce abnormal chromosomal segregation,^43^ and aberrant mitotic events have been linked to the increased activation of autophagy.^75^ TTFields-treated cells that underwent aberrant mitosis (identified as cells displaying abnormal numbers of chromosomes or abnormal cell morphology) were shown to be more likely to activate autophagy in comparison with cells that had not divided over the course of the experiment or cells that underwent normal cell division,^68^ suggesting that TTFields-induced aberrant mitotic events could be driving activation of autophagy. The phenomenon of ‘doryphagy’ describes the specific autophagy-mediated turnover of centrosomal satellites that leads to chromosome-segregation errors and chromosomal instability.^76^ Given the data described above, it would be interesting to determine if TTFields impact on centrosomal proteins through autophagy-mediated degradation.

The phosphatidylinositol 3-kinase (PI3K)/protein kinase B (Akt)/mammalian target of rapamycin (TOR) signalling pathway is known to suppress the activation of autophagy.^77^ Kim et al. found that the expression of Akt2 and the downstream targets of mTOR complex (mTORC)1, 4E-binding protein 1 (4EBP1) and 70-kDa ribosomal protein S6 kinase (p70S6K), was downregulated in glioma cells upon TTFields therapy, and that phosphorylation of mTOR at Ser2448 was reduced. Re-expressing Akt2 prevented the TTFields-mediated induction of autophagy, indicating that Akt2 pathway signalling regulates autophagy in TTFields-treated cells, and that TTFields activate autophagy by suppressing the inhibitory action of PI3K/Akt/mTOR pathway on autophagy.^69^ Additionally, the function of mTORC1 is inhibited by various types of stress within the cell. For example, AMP-dependent kinase (AMPK), which phosphorylates and inhibits mTORC1, thereby suppressing the inhibitory effects of mTORC1 on autophagy,^78^ is activated by low-energy (ATP) levels. Shteingauz et al. demonstrated that intracellular levels of ATP were reduced in surviving cells after TTFields application, and that knockdown of AMPK prevented TTFields-mediated upregulation of autophagy, suggesting that activation of AMPK was required for increased activation of autophagy in TTFields-treated cells.^68^ Together, these data suggest that activation of autophagy in TTFields-treated GBM cells might be mediated via regulation of the PI3K/Akt/mTOR signalling pathway (Fig. 3C).

Whether activation of autophagy by TTFields serves as a cell survival or a cell death signal is still unclear. Some studies have shown that inhibition of autophagy enhances the killing of cancer cells with TTFields, suggesting that upregulation of autophagy might act as a mechanism of resistance to TTFields, and thus highlighting the potential use of autophagy inhibition as a strategy to enhance the therapeutic efficacy.^68^ Others, however, have reported that autophagy inhibition reduces the killing of cancer cells with TTFields.^69^ For example, Silginer et al. reported that TTFields-mediated cell death took place in a caspase-independent manner, and that autophagy played an important role in TTFields-mediated cell death.^70^ However, TTFields-mediated cell death has been shown to occur through both caspase-dependent (characteristic of apoptotic cell death) and caspase-independent pathways,^43,46,70^ suggesting that the type of cell death activated upon TTFields application might be conditional, influenced, for example, by cancer type and genetic context.^70,79^ The regulatory mechanisms that direct autophagy to act as a pro-survival or pro-death signal following TTFields warrant further study, but could facilitate the identification of defined populations of patients that are most likely to benefit from concomitant inhibition of autophagy.

### TTFields and innate immunity

Macrophages play a central role in governing the nature of immune responses, and represent the dominant infiltrating immune cell population in GBM, constituting ~30–40% of the tumour mass.^80^ Macrophages can assume one of two phenotypes: M1 macrophages are considered proinflammatory and secrete proinflammatory cytokines, such as interleukin-1β (IL-1β), IL-12 and tumour necrosis factor-α (TNF-α),^81^ to initiate an immune response; M2 macrophages are involved in the resolution of inflammation and release anti-inflammatory cytokines, including IL-10 and transforming growth factor-β (TGF-β). In addition, macrophages are themselves stimulated by cytokines.^81,82^ Inflammatory cytokines stimulate macrophages to produce nitric oxide (NO), which induces toxic reactions against invading pathogens and regulates the function of host immune cells, such as T cells, antigen-presenting cells, mast cells, neutrophils and natural killer cells. NO is converted from l-arginine by the inducible NO synthase (iNOS) during inflammation. The proinflammatory cytokines TNF-α, IL-1β and IL-6 mediate the upregulation of iNOS in macrophages by activating the nuclear factor κB (NF-κB) transcription factor and the mitogen-activated protein kinase (MAPK) protein p38, extracellular signal-regulated kinase (Erk)1/2 and c-Jun-activated kinase (JNK). Additionally, when macrophages are first activated by cytokines, the low concentrations of NO can stimulate the NF-κB signalling pathway to upregulate iNOS expression in a positive-feedback loop.^83^ Park et al.^84^ showed that the mRNA expression levels of IL-1β and TNF-α were significantly increased in RAW 264.7 murine macrophage cells following TTFields treatment. TTFields therapy also upregulated iNOS at both the mRNA and protein levels, consistent with the increased production of NO in these cells. Additionally, an increase in IL-1β, TNF-α and IL-6 secreted into the medium of TTFields-treated RAW 264.7 cells co-cultured with 4T1 cancer cells was detected. These data indicate that TTFields promote phenotype skewing of macrophages towards a proinflammatory phenotype. Furthermore, 4T1 cells that were exposed to the culture medium from TTFields-treated RAW 264.7 cells displayed a reduction in cell viability compared with 4T1 cells that were exposed to the culture medium of untreated RAW 264.7 cells, suggesting that TTFields-mediated activation of macrophages promotes antitumour immunity.^84^

Reactive oxygen species (ROS), which are also produced by macrophages, function as secondary messengers that activate both NF-κB and MAPK signalling pathways within these cells.^85^ Under normal circumstances, the inhibitor of NF-κB (IκB-α) protein is bound to, and inhibits, NF-κB, sequestering it in the cytoplasm. Both ROS and TNF-α can mediate the activation of MAPK signalling^85^ and IκB kinase (IKK), which phosphorylates IκB-α, resulting in its polyubiquitination and subsequent proteasomal degradation. This process releases the p65 transcriptional subunit from the NF-κB complex, which can then translocate to the nucleus and regulate the transcription of target genes, including those encoding proinflammatory cytokines.^86^ Interestingly, ROS secretion was increased in RAW 264.7 cells following TTFields treatment. Additionally, TTFields-treated RAW 264.7 cells displayed increased phosphorylation of IκB-α, the NF-κB p65 subunit and p38 MAPK.^84^ These data suggest that TTFields therapy mediates its antitumour immunity effects via the regulation of NF-κB and MAPK signalling pathways in RAW 264.7 macrophages, and raises the potential that TTFields could provide a way to overcome the mechanisms of immune escape typically associated with glioblastoma.^87^

### TTFields enhance immunogenic cell death

Using a rabbit model of metastatic cancer, Kirson et al.^88^ showed that applying TTFields therapy to VX-2 cell (squamous cell carcinomas) implanted within the kidney capsule significantly reduced distant metastases in the lungs. As the lungs were not directly treated, the observed abscopal effect was most likely mediated by the immune system. Indeed, there was a significant increase in the infiltration of CD45+ T cells, CD4+ T helper cells and CD8+ cytotoxic T cells to the lungs of the treated rabbits, confirming that TTFields can stimulate antitumour immunity in vivo.^88^

Voloshin et al.^89^ showed that TTFields induce the membrane translocation and subsequent exposure of the chaperone calreticulin, as well as secretion of the damage-associated molecular pattern ATP and high-mobility group box protein 1 (HMGB1), both of which lead to immunogenic death in cancer cells. Furthermore, dendritic cells, when co-cultured with TTFields-treated cells, demonstrated enhanced phagocytic activity and upregulation of the major histocompatibility complex (MHC) class II and the activation markers CD40 and CD80, indicative of enhanced dendritic cell maturation and immunogenic cell death. Notably, combining TTFields with an inhibitor of programmed cell death protein 1 (PD-1) reduced the tumour volume in lung- and colon-tumour-bearing mice compared with sham control and compared with mice treated with TTFields or the inhibitor alone,^89^ suggesting that PD-1 inhibition might further promote the antitumour immune response elicited by TTFields treatment. PD-1 normally functions by disrupting T-cell activation, thereby preventing activation of the immune response; this immune checkpoint, and others, is essential to prevent hyperactivation of the immune system, which can result in autoimmune disorders (e.g., rheumatoid arthritis and multiple sclerosis), but cancer cells can exploit this mechanism in order to evade immune-response-mediated cell death.^90^ Immune checkpoint inhibitors such as anti-PD-1 thus enable T cells to kill cancer cells again and further enhance the anticancer immune response induced by TTFields therapy (Fig. 3D). Several clinical trials assessing the use of immune checkpoint inhibitors in glioblastoma are ongoing, whilst the combination of TTFields with anti-PD-1 therapy is currently being assessed in patients with NSCLC (NCT02973789),^91^ potentially providing an important platform for future clinical studies assessing a similar combination to treat patients with a glioblastoma.

### TTFields suppress cancer cell migration

GBMs are locally invasive tumours. As the extensive migration and infiltration of glioma cells into healthy brain tissue contribute to therapeutic resistance and poor outcomes, restraining these properties represents an appealing therapeutic strategy.^92^ Studies by both Kim et al. and Silginer et al. demonstrated that the application of TTFields therapy to established GBM cells significantly reduced cancer cell migration and invasion, as determined through the use of scratch wound-healing and Transwell systems.^54,70^ Silginer et al. also identified similar anti-migratory effects of TTFields on glioma-initiating cells, which can undergo self-renewal and initiate tumorigenesis; this is an important finding given the key role played by glioma-initiating cells in mediating therapeutic resistance and recapitulating the tumour cell hierarchy following treatment.^93–95^ Interestingly, the results indicated that the anti-infiltrative effects of TTFields were mediated by downregulation of the NF-κB, MAPK and PI3K/AKT signalling pathways in glioma cells, which modulate the transcriptional regulation of matrix metalloproteinase (MMP)2 and MMP9 (Fig. 3E).^54^ Additionally, TTFields were found to reduce the expression of vascular endothelial growth factor (VEGF) and hypoxia-inducible factor (HIF)-1α, and to suppress vascular development using human umbilical vein endothelial cells within an in vitro Matrigel™-based endothelial tube-formation assay.^54^ The ability of glioma-initiating cells to remodel the tumour microenvironment, including via complex interplay with endothelial cells, has been implicated in their aggressive nature.^95^ These findings therefore suggest that TTFields may restrain the invasiveness of GBM by reducing MMP-mediated cleavage through ECM proteins in the surrounding brain by glioma cells and potentially reducing nutrient supply by limiting neovascularisation through reduced VEGF and HIF-1α production in glioma cells.

### TTFields enhance cell membrane permeability and intracellular drug concentrations

Whereas integral membrane proteins mediate the transport of large molecules across the cell membrane by passive or active transport, small molecules and ions can simply diffuse across the cell membrane through small holes that punctuate the surface of the cell membrane.^96^ Chang et al.^97^ used scanning electron microscopy to reveal that TTFields increased both the number and the size of holes in the membrane of glioma cells, with an average hole size of 240.6 ± 91.7 nm^2^ in TTFields-treated cells compared with 129.8 ± 31.9 nm^2^ in untreated cells. Importantly, these changes appear to be cancer-specific, as no changes in the membrane structure of healthy human fibroblast cells were observed. Additionally, the authors observed a significant increase in the uptake of membrane-associating reagents with a size of 20 kDa, and no larger than 50 kDa, into glioma cells with TTFields. These changes were reversible and returned to normal within 24 h of ceasing TTFields treatment^97^ (Fig. 3F). Emerging data also suggest that the application of TTFields therapy might interfere with the integrity of the blood–brain barrier by transiently disrupting the localisation of tight-junction proteins such as claudin 5 and ZO-1.^98^ Additional studies and their results will be highly informative.

These findings suggest that TTFields therapy has the potential to increase intracellular/intratumoral concentrations of chemotherapy, and therefore provides a rational explanation for the reported increase in sensitivity to chemotherapeutic drugs observed following TTFields therapy—perhaps even explaining the significant improvement in patient survival observed in the EF-14 trial when TTFields was combined with TMZ.^16^ Therefore, in theory, TTFields might enhance the clinical efficacy of many pharmacotherapies, independent of drug mechanism, by increasing the drug concentration selectively within neoplastic cells. These studies also highlight important implications for the rational design of TTFields–chemotherapy combinations and drug scheduling, since ensuring TTFields delivered prior to drug administration could help optimise therapeutic response (e.g., exit may be beneficial to delay drug doses until a few hours after scheduled breaks in TTFields therapy).

## An overview and update on TTFields clinical trials

The emerging landscape of clinical trials assessing TTFields therapy to treat intracranial and extracranial tumours has to date supported FDA approvals (and a European CE mark for Optune) for the indications of recurrent and newly diagnosed glioblastoma and mesothelioma (discussed below). Additionally, over 25 registered clinical trials assessing TTFields are currently active.^99^

### Recurrent GBM

From 2006 until 2009, 237 patients with recurrent GBM were enrolled in a randomised Phase 3 clinical trial (EF-11, the first Phase 3 trial to investigate the efficacy of TTFields as a monotherapy in humans) and treated with either TTFields (120 patients) or chemotherapy alone (117 patients). The primary endpoint was OS, and secondary endpoints included progression-free survival (PFS), 1-year survival, quality of life (QoL) and safety/toxicity. Although there was no significant difference in OS or PFS in TTFields-treated patients compared with the chemotherapy control group (6.6 vs 6.0 months and 2.2 vs 2.1 months, respectively), TTFields therapy was concluded to be just as effective as physician’s choice chemotherapy in treating recurrent GBM, with the added benefits of fewer severe adverse events and overall improvement in QoL.^20^ The results of the EF-11 trial led to the FDA approval of TTFields as a treatment for GBM recurrence following standard-of-care chemotherapy.^21^

### Newly diagnosed GBM

Between 2009 and 2014, a randomised, Phase 3 clinical trial (EF-14) enrolled patients to receive either TTFields plus adjuvant TMZ (466 patients) or TMZ alone (229 patients). All patients had completed initial radiotherapy with concomitant TMZ prior to randomisation. The study primary endpoint was PFS. The secondary endpoint was OS, with further exploratory endpoints, including PFS at 6 months, QoL and cognitive function. TTFields with TMZ significantly increased both the PFS and OS of newly diagnosed GBM patients by 2.7 months (6.7 vs 4.0 months, *P* < 0.001) and 4.9 months (20.9 vs 16.0 months, *P* = 0.004), respectively, compared with treatment with TMZ alone. Two years into the trial, 43% of patients randomised to receive TTFields plus TMZ treatment were still alive, compared with 29% in the TMZ-only group. The addition of TTFields to TMZ did not compromise QoL or increase the rate of serious adverse events.^16^ Following the results of the EF-14 trial, the FDA approved TTFields together with concomitant TMZ for the treatment of newly diagnosed GBM after maximal resection and completion of standard-of-care radiotherapy and chemotherapy.^21^

### Brain metastases and extracranial tumours

Clinical trials assessing the impact of TTFields to treat brain metastases from NSCLC and a range of other extracranial malignancies (including mesothelioma, NSCLC and pancreatic, ovarian, hepatic and gastric cancer) are actively progressing. These trials are summarised in Table 1. Although it is not possible to provide a detailed commentary on each trial within this review, the results of the STELLAR trial, completed in 2019, should be highlighted.^100^ This single-arm Phase 2 clinical trial examined the safety and efficacy of continuous TTFields delivery (>18 h per day) at 150 kHz in combination with standard-of-care chemotherapy to treat patients with unresectable treatment-naive malignant pleural mesothelioma. The trial demonstrated encouraging median OS and PFS of 18.2 and 7.6 months, respectively, which are considered to represent a major advance compared with OS and PFS of 12.1 and 5.7 months for historical controls receiving standard-of-care chemotherapy only.^101^ In light of the STELLAR trial, TTFields received FDA approval for use in combination with chemotherapy to treat malignant pleural mesothelioma under humanitarian device exemption^102^ (a regulatory framework which helps facilitate device approval for rare diseases; this recognises the challenge of generating clinical evidence with a limited patient population, and whilst stringent safety criteria must be maintained, the device can be exempt from some effectiveness requirements, subject to certain profit and use restrictions). This approval established TTFields therapy as the first FDA-approved mesothelioma treatment in over 15 years. A summary of key completed and ongoing TTFields clinical trials is detailed in Table 1.

**Table 1:** Key TTFields clinical trials—completed and ongoing.

Intracranial tumours						
Indication & trial ^(ref.)^	Status & design	*n* (rec dates)	Treatment(s)	1° Endpoint	2° Endpoint(s)	Remarks/conclusions
rGBM EF-11^20^ *NCT00379470*	COMPLETE Phase 3 Randomised	237 patients (2006–2009)	(A) TTFields alone (120) (B) Chemo alone (117)	OS: 6.6 m (TTFields) vs 6.0 m N/S	(A) PFS: 2.2 m (TTFields) vs 2.1 m N/S. (B) OS at 1 year: 20% in both groups. (C) Toxicity: 6% (TTFields) vs 16% (chemo) AEs^(G3–4)^ (*P* = 0.022). Confirmed TTFields-related AEs = 2% (Grade 2 skin AEs). (D) QoL: ↑ cognitive & emotional, slight ↓ physical functioning.	• TTFields as effective (OS/PFS) as physician’s choice chemotherapy. • ↓ Severe AEs and overall ↑ QoL. • Led to FDA approval of TTFields for rGBM.^21^
nGBM EF-14^16^ *NCT00916409*	COMPLETE Phase 3 Randomised	695 patients (2009–2014)	(A) TTFields + TMZ (466) (B) TMZ alone (229)	PFS: 6.7 m (TTFields + TMZ) vs 4.0 m (*P* < 0.2006)	(A) OS: 20.9 m (TTFields + TMZ) vs 16 m (*P* < 0.001). (B) Toxicity: 48% (TTFields + TMZ) vs 44% AEs^(G3–4)^—TTFields-related = 2% (all grade 3 skin). (C) Tolerability: sig. delay in 6-pt MMSE decline and 10-pt KPS decline.	• Randomisation after initial radiochemotherapy. • Patients alive at 5 y post randomisation = 13% (TTFields + TMZ) vs 5% (*P* = 0.004). • TTFields improved survival with no added toxicity or compromise in QoL. • Led to FDA approval for TTFields with TMZ for nGBM following maximal surgical debulking and SoC RT with concomitant TMZ.^21^
nGBM TRIDENT *NCT# pending*	In registration Phase 3 Randomised	950 patients— planned (2020 + )	(A) Concomitant TTFields + RT + TMZ (B) RT + TMZ	OS	(A) PFS (median and at 2, 6 and 12 m). (B) OS at 1 and 2 years. (C) Radiological response (ORR, RANO). (D) Toxicity (AEs). (E) QoL (EORTC QLQ C30). (F) Pathological Δ in resected rGBM post treatment.	• Study to establish the efficacy and safety of 200-kHz TTFields in combination with RT (and TMZ) in patients with nGBM. • Builds on preclinical work suggesting synergy between TTFields and RT.^55,115,116^ • RT delivered through the TTFields arrays to maximise device usage and the putative radiosensitising effects of TTFields.
Brain metastases (1–10 from NSCLC origin) METIS^118^ *NCT02831959*	Ongoing (recruiting) Phase 3 Randomised	260 patients— planned (2016–2020)	(A) TTFields after SRS (130 planned) (B) SRS only (130 planned)	Time to first intracranial progression	(A) OS. (B) Toxicity (AEs). (C) QoL. (D) Radiological response (RANO-BM & RECIST V1.1). (E) Neurocognitive failure.	• Study to establish the efficacy and safety of 150-kHz TTFields in patients with brain metastases from NSCLC. • Builds on Phase 1/2 trial of TTFields for primary lesion in advanced NSCLC.^119^ • Estimated study completion: Dec 2020.

Extracranial tumours						
Indication & trial ^(ref.)^	Status & design	*n* (rec dates)	Treatment(s)	1° Endpoint	2° Endpoint(s)	Remarks/conclusions
Mesothelioma STELLAR^100,120^ *NCT02397928*	COMPLETE Phase 2 Single arm	80 patients (2015–2017)	TTFields + chemo (pemetrexed with cisplatin or carboplatin)	OS: 18.2 m	(A) PFS: 7.6 m (B) Toxicity: 36% AEs^(G3–4)^ + 3 (4%) chemotherapy-related deaths—TTFields-related = 5% (all grade 3 skin). (C) Radiological response (mRECIST): 40% partial response, 57% stable disease at first F/U scan (6 weeks).	• Study aimed to identify a signal for the activity of 150 kHz TTFields with chemotherapy in patients with unresectable malignant pleural mesothelioma. • Encouraging survival data compared with historical OS of 12.1m.^101^ • Led to first FDA-approved mesothelioma treatment in over 15 years.^102^
NSCLC ^119^ *NCT00749346*	COMPLETE Phase 1/2 Single arm	42 patients (2008–2009)	TTFields + chemo (pemetrexed)	Time to local ('in-field') progression: 28w	(A) (Systemic) PFS: 22 weeks. (B) OS: 13.8 m (57% survival at 1 year) (C) Radiological response: 15% partial response, 49% stable disease. (D) Toxicity: no TTFields-related SAEs.	• Study to establish the safety and potential efficacy of TTFields in patients with NSCLC with advanced disease following first-line therapy and eligible for second line pemetrexed. • Encouraging survival relative to historical control for pemetrexed only^121^ (2.9 m historic vs 5 m+ with TTFields). • TTFields + pemetrexed is safe and potentially effective—led to Phase 3 LUNAR trial (below).
NSCLC LUNAR^91^ *NCT02973789*	Ongoing (recruiting) Phase 3 Randomised	534 patients— planned (2016–2021)	(A) TTFields + ICI or DOCE (B) ICI or DOCE	OS (TTFields + ICI/DOCE vs ICI/DOCE alone)	(A) OS (TTFields + ICI vs ICI) and OS (TTFields + DOCE vs DOCE). (B) PFS. (C) Toxicity (AEs). (D) Radiological response. (E) QoL (EORTC QLQ C30 questionnaire). (F) Exploratory non-inferiority analysis of TTFields+DOCE vs ICI.	Study to establish whether the addition of TTFields to ICI or docetaxel with improve OS in stage IV NSCLC following platinum doublet failure. • Estimated study completion: December 2021.
Pancreatic cancer PANOVA ^122^ *NCT01971281*	COMPLETE Phase 2 Non-randomised	40 patients (2013–2017)s	(A) TTFields + GEM (B) TTFields + GEM & nab-P* *Arm added to reflect new SoC established while trial was ongoing*.^123^	Toxicity (SAEs): TTFields+GEM = 85% TTFields+GEM + nab-P = 85% (individual rates similar to historic non-TTFields control^123^) G3 TTFields-related skin toxicities = 18%	(A) OS: 14.9 m (TTFields + GEM) & median OS not reached (>15 m) (TTFields + GEM + nab-P). (B) PFS: 8.3 m (TTFields + GEM) & 12.7 m (TTFields + GEM + nab-P)	• Study to establish the safety and preliminary efficacy of TTFields with chemotherapy in patients with pancreatic ductal adenocarcinoma. • One-year OS rates of 55% (TTFields + GEM) & 72% (TTFields + GEM + nab-P)—encouraging relative to historic control 22% (GEM) & 35% (GEM + nab-P).^123^ • TTFields+GEM + /-nab-P represents a safe, potentially effective combination—led to Phase 3 PANOVA-3 trial (below).
Pancreatic cancer PANOVA-3^124^ *NCT03377491*	Ongoing (recruiting) Phase 3 Randomised	556 patients— planned (2017–2022)	(A) TTFields + GEM & nab-P (B) GEM & nab-P	OS	(A) PFS. (B) Toxicity (AEs). (C) Radiological response. (D) Resectability rate. (E) QoL.	• Study to establish efficacy of TTFields with standard-of-care GEM & nab-P to treat patients with unresectable, locally advanced pancreatic adenocarcinoma. • Estimated study completion: December 2022.
Ovarian cancer INNOVATE (EF-22)^125^ *NCT02244502*	COMPLETE Phase 2 Single arm	31 patients (2014–2016)	TTFields + PAC	Toxicity: G3–4 AEs 55% patients – no increase relative to historic non-TTFields control. G3 TTFields-related skin toxicities = 6%.	(A) OS: median not reached (>21 m). (B) PFS: 8.9 m. (C) Response rate: 25% partial response, 46% stable disease.	• Study to establish the safety and preliminary efficacy of TTFields (200 kHz) with PAC to treat patients with recurrent, platinum-resistant ovarian carcinoma. • Heavily pre-treated patient cohort— median 4 prior chemotherapy lines and 2 lines on/after platinum at enrolment. • Encouraging median OS relative to previous studies inc. 17.6 m, 11.3 m and 6.2 m after first, second and fourth relapse by Hanker et al.^126^ • Led to Phase 3 INNOVATE-3 trial (below).
Ovarian cancer INNOVATE-3^127^ *NCT03940196*	Ongoing (recruiting) Phase 3 Randomised	540 patients— planned (2019–2024)	(A) TTFields + PAC (B) PAC	OS	(A) PFS. (B) Toxicity (AEs). (C) Radiological response rate. (D) QoL (EORTC QLQ C30).	• Study to establish efficacy of TTFields with standard-of-care PAC to treat patients with platinum-resistant ovarian cancer within 6 months of last platinum therapy with ≤2 previous lines following diagnosis of PROC and maximum total of 5 prior lines of systemic therapy. • Estimated study completion: December 2024.
Hepatocellular carcinoma HEPANOVA^128^ *NCT03606590*	Ongoing (recruiting) Phase 2 Single arm	25 patients – planned (2019–2020)	TTFields + sorafenib	Overall response rate (patients with CR or PR as per RECIST criteria)	(A) OS. (B) PFS. (C) In-field control rate at 1 year. (D) Distant metastases-free survival at 1 year.	• Study to establish the safety and preliminary efficacy of adding TTFields (150 kHz) to sorafenib in patients with unresectable HCC. • Estimated study completion: December 2020.
Gastric cancer *NCT04281576*	Ongoing (recruiting) Phase 2 Single arm	28 patients – planned (2019–2022)	TTFields + XELOX (+ Trastuzumab if HER-2 positive)	Overall response rate	(A) OS. (B) PFS. (C) Disease control rate. (D) Time to progression. (E) Duration of response. (F) 12-month OS rate. (G) Toxicity (AEs).	• Study to establish the safety and preliminary efficacy of TTFields (150 kHz) with SoC chemotherapy as first-line treatment for patients with unresectable, locally advanced or metastatic gastroesophageal junction or gastric adenocarcinoma. • Estimated study completion: December 2022.

*rGBM* recurrent GBM, *nGBM* newly diagnosed GBM, *NSCLC*   non-small-cell lung cancer, *TTFields* tumour-treating fields, *TMZ*   temozolomide, *SoC* standard of care, *DOCE*   docetaxel, *GEM*   gemcitabine, *nab-P*   nab-paclitaxel, *PAC*   paclitaxel, *ICI*   immune checkpoint inhibitors, *XELOX*   capecitabine (Xeloda) + oxaliplatin (Eloxatin), *RT*   radiotherapy, *SRS*   stereotactic radiosurgery, *OS*   (median) overall survival, *PFS*   (median) progression-free survival, *CR*   complete response, *PR*   partial response, *ORR*   overall response rate (proportion of patients with a PR or CR), *F/U*   follow-up, *QoL* quality of life, *AEs* adverse events, *AEs*^*(G3–4)*^ grade 3–4 adverse events, *SAEs* severe adverse events, *MMSE*   mini-mental-state examination, *KPS*   Karnofsky performance score, *N/S*   no significant difference.

## Conclusions and future opportunities

Further potential applications for TTFields include expanding the population of patients receiving this therapy through additional trials for which existing and emerging preclinical data support clinical studies (e.g., colorectal, renal and breast). In addition, investigation of cancers that have not yet been studied in the context of TTFields—haematological cancers, for example—could be considered. Several key ongoing clinical trials (see Table 1) evaluating the efficacy of combining TTFields with existing anticancer agents in a range of cancer types together with increasing mechanistic data derived from preclinical studies will help to clarify the role of TTFields in treatment regimens and establish the feasibility of making TTFields more readily available across a wider range of cancer types in the years to come. As cancers from different anatomical regions gain attention, transducer array redesigns might be required to ensure optimal TTFields delivery in new anatomical regions whilst maintaining QoL.

Solid paediatric malignancies, including brain tumours, represent a clear indication in need of less harmful anticancer therapies.^103^ Ongoing preclinical research suggests that TTFields might demonstrate efficacy in paediatric GBM, medulloblastoma and ependymoma,^104^ whilst limited case reports/series suggest that TTFields treatment in children is likely to be safe.^105,106^ One of these studies indicated that TTFields was tolerable in five paediatric patients with high-grade glioma, aged between 10 and 20, three of whom showed partial responses when they received TTFields alongside chemotherapy and/or radiation.^106^ The study of the use of TTFields to improve outcomes and avoid the substantial morbidity associated with use of chemotherapy and radiotherapy in children is an area of investigation that should be prioritised. To this end, an investigator-initiated study (NCT03033992) testing the feasibility of TTFields for children with recurrent or progressive supratentorial high-grade glioma and ependymoma is ongoing; additional studies and long-term follow-up data would, however, be desirable.

Regarding the treatment of adult patients with newly diagnosed GBM, even with improvements in outcome following the addition of TTFields, survival for most patients remains under 2 years,^16^ highlighting an ongoing need to further enhance the efficacy of TTFields and current chemoradiotherapy. The EF-14 trial concluded that compliance with TTFields therapy correlated with improved OS in GBM patients. Patients with a compliance of over 90% (≥ 18 h of daily TTFields) had a median OS of 28.7 months from diagnosis and a 5-year survival rate of 29.3%,^7^ and simulation-based analysis of the EF-14 Phase 3 data suggests that the overall dose of TTFields delivered at the tumour site strongly correlates with OS.^107^ Considering these findings, it is clear that any method that could effectively increase TTFields usage time (such as improved portability of the clinical device^108^) or increase intensity at target regions should support improvements in therapeutic efficacy.

Emerging preclinical data outlined in this review suggest a strong mechanistic rationale for the use of TTFields in combination with a number of molecularly targeted therapies to improve efficacy. Preclinical data have shown that PD-1 inhibitors can increase antitumour immunity with TTFields^89^ and underpin a currently ongoing clinical trial investigating the efficacy of combining PD-1 inhibitors with TTFields for the treatment of patients with NSCLC (LUNAR NCT02973789—see Table 1). A state of ‘BRCAness’ (deficiency in BRCA or related HRR function) following TTFields-mediated downregulation of BRCA genes (discussed above) has been described.^53,55^ BRCA-deficient cancers are characterised by an inherent vulnerability to DNA single-strand break repair inhibitors, such as PARP inhibitors,^109,110^ under the concept of synthetic lethality due to impaired HRR efficiency.^111^ Preclinical data have shown TTFields and olaparib, a PARP1 inhibitor, synergised to increase cell killing compared with either treatment alone.^61^ Future trials combining TTFields with PARP inhibition, including an active trial (recruitment commenced in early 2020) using niraparib and TTFields in GBM (NCT04221503), will be highly informative. The combination of TTFields with other DNA-damage response processes should also be considered. In this respect, our team are actively investigating TTFields combinations that incorporate inhibition of the Fanconi anaemia pathway, as this pathway has been implicated in therapeutic resistance to TMZ^112–114^ and also demonstrates synthetic lethality with loss of BRCA.^60^

TTFields-induced ‘BRCAness’ could also enhance the response to radiotherapy. This effect has been demonstrated in a number of preclinical research projects.^55,115,116^ It could, therefore, be hypothesised that the simultaneous delivery of TTFields with chemoradiotherapy in the clinic should maximise the DNA-damage-response-modulating influence of TTFields and might lead to a synergistic effect. Encouragingly, a pilot study of TTFields concomitant with radiotherapy and TMZ in ten newly diagnosed GBM patients demonstrated that this regimen does not increase toxicity.^117^ This study is being expanded in a Phase 2 clinical trial (NCT03869242), and a similar Phase 3 trial is currently in registration (TRIDENT—see Table 1). Finally, future studies should also aim to identify and characterise predictive biomarkers that could help to identify which patients most likely benefit from TTFields treatment.

As evident from the preclinical and clinical studies highlighted in this review, TTFields has great potential, both in the short- and long term, to improve outcomes for many patients diagnosed with a range of cancers. Continuing to enhance our knowledge of the molecular mechanisms that underpin TTFields-based cellular toxicity and tumour specificity/therapeutic index will hopefully aid further adoption of this new modality and integration into existing and novel treatment strategies to improve outcomes for a wide range of cancer patient cohorts.

## Data Availability

Not applicable.

## References

[1] Philips A, Henshaw DL, Lamburn G, O’Carroll MJ (2018). Brain tumours: rise in glioblastoma multiforme incidence in England 1995-2015 suggests an adverse environmental or lifestyle factor. J. Environ. Public Health v..

[2] Patel, A. P., Fisher, J. L., Nichols, E., Abd-Allah, F., Abdela, J., Abdelalim, A., Abraha, H. N., Agius, D., Alahdab, F., Alam, T. & Allen, C.A. Global, regional, and national burden of brain and other CNS cancer, 1990-2016: a systematic analysis for the Global Burden of Disease Study 2016. *Lancet Neurol*. **18**, 376–393 (2019). 10.1016/S1474-4422(18)30468-X PMC6416167 30797715

[3] Alexander BM, Ba S, Berger MS, Berry DA, Cavenee WK, Chang SM (2018). Adaptive global innovative learning environment for glioblastoma: GBM AGILE. Clin. Cancer Res..

[4] Ostrom QT, Cote DJ, Ascha M, Kruchko C, Barnholtz-Sloan JS (2018). Adult glioma incidence and survival by race or ethnicity in the United States From 2000 to 2014. JAMA Oncol..

[5] Stupp R, Hegi ME, Mason WP, van den Bent MJ, Taphoorn MJ, Janzer RC (2009). Effects of radiotherapy with concomitant and adjuvant temozolomide versus radiotherapy alone on survival in glioblastoma in a randomised phase III study: 5-year analysis of the EORTC-NCIC trial. Lancet Oncol..

[6] Cancer Research UK. Tackle cancers with substantial unmet need: our research strategy. http://www.cancerresearchuk.org/funding-for-researchers/our-research-strategy/tackle-cancers-with-substantial-unmet-need (2017).

[7] Toms S, Kim C, Nicholas G, Ram Z (2019). Increased compliance with tumor treating fields therapy is prognostic for improved survival in the treatment of glioblastoma: a subgroup analysis of the EF-14 phase III trial. J. Neuro-Oncol..

[8] Kirson ED, Dbalý V, Tovarys F, Vymazal J, Soustiel JF, Itzhaki A (2007). Alternating electric fields arrest cell proliferation in animal tumor models and human brain tumors. Proc. Natl Acad. Sci. USA.

[9] Kirson ED, Gurvich Z, Schneiderman R, Dekel E, Itzhaki A, Wasserman Y (2004). Disruption of cancer cell replication by alternating electric fields. Cancer Res..

[10] Moghadam M, Firoozabadi S, Janahmadi M (2008). 50 Hz alternating extremely low frequency magnetic fields affect excitability, firing and action potential shape through interaction with ionic channels in snail neurones. Environmentalist.

[11] Cheung AY, Neyzari A (1984). Deep local hyperthermia for cancer therapy: external electromagnetic and ultrasound techniques. Cancer Res..

[12] Davies AM, Weinberg U, Palti Y (2013). Tumor treating fields: a new frontier in cancer therapy. Ann. N. Y. Acad. Sci..

[13] Eilon DK, Vladimír D, František T, Josef V, Jean FS, Aviran I (2007). Alternating electric fields arrest cell proliferation in animal tumor models and human brain tumors. Proc. Natl Acad. Sci. USA.

[14] Cohen, M. H., Johnson, J. R. & Pazdur, R. Food and drug administration drug approval summary: temozolomide plus radiation therapy for the treatment of newly diagnosed glioblastoma multiforme. *Clin. Cancer Res.* **11**, 6767–6771 (2005). 10.1158/1078-0432.CCR-05-0722 16203762

[15] Kesari S, Ram Z (2017). Tumor-treating fields plus chemotherapy versus chemotherapy alone for glioblastoma at first recurrence: a post hoc analysis of the EF-14 trial. CNS Oncol..

[16] Stupp R, Taillibert S, Kanner A, Read W, Steinberg DM, Lhermitte B (2017). Effect of tumor-treating fields plus maintenance temozolomide vs maintenance temozolomide alone on survival in patients with glioblastoma: a randomized clinical trial. J. Am. Med. Asooc..

[17] Stupp R, Taillibert S, Kanner AA, Kesari S, Steinberg DM, Toms SA (2015). Maintenance therapy with tumor-treating fields plus temozolomide vs temozolomide alone for glioblastoma: a randomized clinical trial. J. Am. Med. Assoc..

[18] Zhu JJ, Demireva P, Kanner AA, Pannullo S, Mehdorn M, Avgeropoulos N (2017). Health-related quality of life, cognitive screening, and functional status in a randomized phase III trial (EF-14) of tumor treating fields with temozolomide compared to temozolomide alone in newly diagnosed glioblastoma. J. Neuro-Oncol..

[19] Mrugala MM, Engelhard HH, Dinh Tran D, Kew Y, Cavaliere R, Villano JL (2015). Clinical practice experience with NovoTTF-100A system for glioblastoma: The Patient Registry Dataset (PRiDe). Semin. Oncol..

[20] Stupp R, Wong ET, Kanner AA, Steinberg D, Engelhard H, Heidecke V (2012). NovoTTF-100A versus physician’s choice chemotherapy in recurrent glioblastoma: a randomised phase III trial of a novel treatment modality. Eur. J. Cancer.

[21] US Food and Drug Administration. Premarket Approval (PMA): Optune. https://www.accessdata.fda.gov/scripts/cdrh/cfdocs/cfpma/pma.cfm?id=P100034S013 (2020).

[22] Chaudhry A, Benson L, Varshaver M, Farber O, Weinberg U, Kirson E (2015). NovoTTF (TM)-100A system (tumor treating fields) transducer array layout planning for glioblastoma: a NovoTAL (TM) system user study. World J. Surg. Oncol..

[23] Lacouture M, Davis ME, Elzinga G, Butowski N, Tran D, Villano J (2013). Dermatologic event characteristics and management with the novoTTF-100A system, a novel anti-mitotic device for the treatment of recurrent glioblastoma (rGBM). Neuro. Oncol..

[24] Novocure. Form S-1 registration statement under the Securities Act of 1933: Novocure Limited: United States Securities and Exchange Commission. https://www.sec.gov/Archives/edgar/data/1645113/000119312515308245/d940664ds1.htm (2015).

[25] Novocure. Patient Information and Operation Manuel. https://www.optune.com/content/pdfs/Optune_PIOM_8.5x11.pdf (2019).

[26] William W, Yeun MiY, Ashley K, Martin C, Marjolaine G-L, Patrick G-S (2018). Assessment of costs associated with adverse events in patients with cancer. PLoS ONE.

[27] Bernard-Arnoux F, Lamure M, Ducray F, Aulagner G, Honnorat J, Armoiry X (2016). The cost-effectiveness of tumor-treating fields therapy in patients with newly diagnosed glioblastoma. Neuro. Oncol..

[28] Connock M, Auguste P, Dussart C, Guyotat J, Armoiry X (2019). Cost-effectiveness of tumor-treating fields added to maintenance temozolomide in patients with glioblastoma: an updated evaluation using a partitioned survival model. J. Neuro-Oncol..

[29] Guzauskas GF, Pollom EL, Stieber VW, Wang BCM, Garrison LP (2019). Tumor treating fields and maintenance temozolomide for newly-diagnosed glioblastoma: a cost-effectiveness study. J. Med. Econ..

[30] Porter KR, McCarthy BJ, Berbaum ML, Davis FG (2011). Conditional survival of all primary brain tumor patients by age, behavior, and histology. Neuroepidemiology.

[31] Larkin J, Hatswell AJ, Nathan P, Lebmeier M, Lee D (2015). The predicted impact of ipilimumab usage on survival in previously treated advanced or metastatic melanoma in the UK. PLoS ONE.

[32] The National Institute for Health and Care Excellence. Ipilimumab for previously treated advanced (unresectable or metastatic) melanoma: guidance and guidelines (TA268). https://www.nice.org.uk/guidance/ta268 (2012).

[33] McCabe C, Claxton K, Culyer A (2008). The NICE cost-effectiveness threshold. Pharmacoeconomics.

[34] Taylor C, Jan S (2017). Economic evaluation of medicines. Aust. Prescr..

[35] Novocure. Novocure reports 2015 operating statistics and financial results. https://www.novocure.com/novocure-reports-2015-operating-statistics-and-financial-results/ (2016).

[36] Novocure. Novocure reports fourth quarter and full year 2019 financial results and provides company update. https://www.novocure.com/novocure-reports-fourth-quarter-and-full-year-2019-financial-results-and-provides-company-update/ (2020).

[37] Novocure. Novocure reports fourth quarter and full year 2018 financial results and provides company update. https://www.novocure.com/novocure-reports-fourth-quarter-and-full-year-2018-financial-results-and-provides-company-update/ (2019).

[38] Mehta M, Wen P, Nishikawa R, Reardon D, Peters K (2017). Critical review of the addition of tumor treating fields (TTFields) to the existing standard of care for newly diagnosed glioblastoma patients. Crit. Rev. Oncol. Hemat..

[39] Wick W (2016). TTFields: where does all the skepticism come from?. Neuro. Oncol..

[40] The National Institute for Health and Care Excellence. Brain tumours (primary) and brain metastases in adults NICE guideline [NG99]. https://www.nice.org.uk/guidance/ng99 (2018). 31393680

[41] Lavy Shahaf G, Giladi M, Schneiderman R, Kinzel A, Weinberg U, Kirson E (2018). P04.17 cancer cell lines response to tumor treating fields: results of a meta-analysis. Neuro. Oncol..

[42] Kline-Smith S, Walczak CE (2004). Mitotic spindle assembly and chromosome segregation: Refocusing on microtubule dynamics. Mol. Cell..

[43] Moshe G, Rosa SS, Tali V, Yaara P, Mijal M, Roni B (2015). Mitotic spindle disruption by alternating electric fields leads to improper chromosome segregation and mitotic catastrophe in cancer cells. Sci. Rep..

[44] Joshua JT, Jordane P, Jack AT, Eric TW (2018). Tubulin’s response to external electric fields by molecular dynamics simulations. PLoS ONE.

[45] Andrea M, Kevin HG (2002). The spindle checkpoint: structural insights into dynamic signalling. Nat. Rev. Mol. Cell Biol..

[46] Nidhi G, Aaron Y, Talia SH, Sze Xian L, Eric TW, Kenneth DS (2015). Tumor treating fields perturb the localization of septins and cause aberrant mitotic exit. PLoS ONE.

[47] Kessler AF, Frömbling GE, Gross F, Hahn M, Dzokou W, Ernestus R-I (2018). Effects of tumor treating fields (TTFields) on glioblastoma cells are augmented by mitotic checkpoint inhibition. Cell Death Dis..

[48] Schulze VK, Klar U, Kosemund D, Wengner AM, Siemeister G, Stöckigt D (2020). Treating cancer by spindle assembly checkpoint abrogation: discovery of two clinical candidates, BAY 1161909 and BAY 1217389, targeting MPS1 kinase. J. Med. Chem..

[49] Field CM, Coughlin M, Doberstein S, Marty T, Sullivan W (2005). Characterization of anillin mutants reveals essential roles in septin localization and plasma membrane integrity. Development.

[50] Spiliotis ET, Kinoshita M, Nelson WJ (2005). A mitotic septin scaffold required for mammalian chromosome congression and segregation. Science.

[51] Paul F, Eric H, Michael L, Mena K, Paknoosh P, Alisa P (2012). An anillin-Ect2 complex stabilizes central spindle microtubules at the cortex during cytokinesis. PLoS ONE.

[52] Goldbach P, Wong R, Beise N, Sarpal R, Trimble WS, Brill JA (2010). Stabilization of the actomyosin ring enables spermatocyte cytokinesis in Drosophila. Mol. Biol. Cell.

[53] Giladi M, Munster M, Schneiderman RS, Voloshin T, Porat Y, Blat R (2017). Tumor treating fields (TTFields) delay DNA damage repair following radiation treatment of glioma cells. Radiat. Oncol..

[54] Kim E, Song H, Yoo S, Yoon M (2016). Tumor treating fields inhibit glioblastoma cell migration, invasion and angiogenesis. Oncotarget.

[55] Karanam NK, Srinivasan K, Ding L, Sishc B, Saha D, Story MD (2017). Tumor-treating fields elicit a conditional vulnerability to ionizing radiation via the downregulation of BRCA1 signaling and reduced DNA double-strand break repair capacity in non-small cell lung cancer cell lines. Cell Death Dis..

[56] Davies AA, Masson J-Y, McIlwraith MJ, Stasiak AZ, Stasiak A, Venkitaraman AR (2001). Role of BRCA2 in control of the RAD51 recombination and DNA repair protein. Mol. Cell.

[57] Scully R, Chen J, Plug A, Xiao Y, Weaver D, Feunteun J (1997). Association of BRCA1 with Rad51 in mitotic and meiotic cells. Cell.

[58] Venkitaraman AR (2001). Functions of BRCA1 and BRCA2 in the biological response to DNA damage. J. Cell Sci..

[59] Mason JM, Chan YL, Weichselbaum RW, Bishop DK (2019). Non-enzymatic roles of human RAD51 at stalled replication forks. Nat. Commun..

[60] Kais, Z., Rondinelli, B., Holmes, A., O’leary, C., Kozono, D, D’andrea Alan d. et al. FANCD2 maintains fork stability in BRCA1/2-deficient tumors and promotes alternative end-joining DNA repair. *Cell Rep.* **15**, 2488–2499 (2016). 10.1016/j.celrep.2016.05.031 PMC4939765 27264184

[61] Karanam, N. K., Hao-Ding, L., Aroumougame, A. & Story, M. D. Tumor treating fields cause replication stress and interfere with DNA replication fork maintenance: implications for cancer therapy. *Transl. Res*. **217**, 33–46 (2020). 10.1016/j.trsl.2019.10.003 31707040

[62] Quinet A, Carvajal-Maldonado D, Lemacon D, Vindigni A (2017). DNA fiber analysis: mind the gap!. Method. Enzymol..

[63] Luke AY, Ricardo JA, Nilisha P, Colleen CC, Joshua AK, Rajika LP (2018). A structural and dynamic model for the assembly of replication protein A on single-stranded DNA. Nat. Commun..

[64] Belotserkovskii BP, Tornaletti S, D’souza AD, Hanawalt PC (2018). R-loop generation during transcription: formation, processing and cellular outcomes. DNA Repair.

[65] Pearl LH, Schierz AC, Ward SE, Al-Lazikani B, Pearl FM (2015). Therapeutic opportunities within the DNA damage response. Nat. Rev. Cancer.

[66] Rominiyi O, Gomez-Roman N, Lad N, Al-Tamimi Y, Jellinek D, Chalmers A (2018). Preclinical evaluation of combinations targeting the DNA damage response in 2D and 3D models of glioblastoma stem cells [abstract]. Neuro. Oncol..

[67] Yun CW, Lee SH (2018). The roles of autophagy in cancer. Int. J. Mol. Sci..

[68] Shteingauz A, Porat Y, Voloshin T, Schneiderman RS, Munster M, Zeevi E (2018). AMPK-dependent autophagy upregulation serves as a survival mechanism in response to tumor treating fields (TTFields). Cell Death Dis..

[69] Kim EH, Jo Y, Sai S, Park MJ, Kim JY, Kim JS (2019). Tumor-treating fields induce autophagy by blocking the Akt2/miR29b axis in glioblastoma cells. Oncogene.

[70] Silginer M, Weller M, Stupp R, Roth P (2017). Biological activity of tumor-treating fields in preclinical glioma models. Cell Death Dis..

[71] Tanida I, Ueno T, Kominami E (2008). LC3 and autophagy. Methods Mol. Biol..

[72] Saori RY, Noboru M (2017). Monitoring and measuring autophagy. Int. J. Mol. Sci..

[73] Orhon, I. & Reggiori, F. Assays to monitor autophagy progression in cell cultures. *Cells*. **6**, 20 (2017). 10.3390/cells6030020 PMC5617966 28686195

[74] Mauthe M, Orhon I, Rocchi C, Zhou X, Luhr M, Hijlkema K-J (2018). Chloroquine inhibits autophagic flux by decreasing autophagosome-lysosome fusion. Autophagy.

[75] Inoue T, Nakayama Y, Li Y, Matsumori H, Takahashi H, Kojima H (2014). SIRT 2 knockdown increases basal autophagy and prevents postslippage death by abnormally prolonging the mitotic arrest that is induced by microtubule inhibitors. FEBS J..

[76] Holdgaard SG, Cianfanelli V, Pupo E, Lambrughi M, Lubas M, Nielsen JC (2019). Selective autophagy maintains centrosome integrity and accurate mitosis by turnover of centriolar satellites. Nat. Commun..

[77] Paquette M, El-Houjeiri L, Pause A (2018). mTOR pathways in cancer and autophagy. Cancers.

[78] Garcia D, Shaw RJ (2017). AMPK: mechanisms of cellular energy sensing and restoration of metabolic balance. Mol. Cell.

[79] Gera N, Yang A, Holtzman TS, Lee SX, Wong ET, Swanson KD (2015). Tumor treating fields perturb the localization of septins and cause aberrant mitotic exit. PLoS ONE.

[80] Chen Z, Hambardzumyan D (2018). Immune microenvironment in glioblastoma subtypes. Front. Immunol..

[81] Wang N, Liang H, Zen K (2014). Molecular mechanisms that influence the macrophage m1-m2 polarization balance. Front. Immunol..

[82] Arango Duque G, Descoteaux A (2014). Macrophage cytokines: involvement in immunity and infectious diseases. Front. Immunol..

[83] Tripathi, P., Tripathi, P., Kashyap, L. & Singh, V. The role of nitric oxide in inflammatory reactions. *FEMS Immunol.**Med. Microbiol.* **51**, 443-452 (2007). 10.1111/j.1574-695X.2007.00329.x 17903207

[84] Park J-I, Song K-H, Jung S-Y, Ahn J, Hwang S-G, Kim J (2019). Tumor-treating fields induce RAW264.7 macrophage activation via NK-κB/MAPK signaling pathways. Technol. Cancer Res. T.

[85] Tan H-Y, Wang N, Li S, Hong M, Wang X, Feng Y (2016). The reactive oxygen species in macrophage polarization: reflecting its dual role in progression and treatment of human. Dis. Oxid. Med..

[86] Ting L, Lingyun Z, Donghyun J, Shao-Cong S (2017). NF-κB signaling in inflammation. Signal Transduct. Tar..

[87] Brown NF, Carter TJ, Ottaviani D, Mulholland P (2018). Harnessing the immune system in glioblastoma. Br. J. Cancer.

[88] Kirson ED, Giladi M, Gurvich Z, Itzhaki A, Mordechovich D, Schneiderman RS (2009). Alternating electric fields (TTFields) inhibit metastatic spread of solid tumors to the lungs. Clin. Exp. Metastasis.

[89] Voloshin T, Kaynan N, Davidi S, Porat Y, Shteingauz A, Schneiderman RS (2020). Tumor-treating fields (TTFields) induce immunogenic cell death resulting in enhanced antitumor efficacy when combined with anti-PD-1 therapy. Cancer Immunol. Immun..

[90] Jiang X, Wang J, Deng X, Xiong F, Ge J, Xiang B (2019). Role of the tumor microenvironment in PD-L1/PD-1-mediated tumor immune escape. Mol. Cancer.

[91] Weinberg, U., Farber, O., Giladi, M., Bomzon, Z. & Kirson, E. Tumor treating fields (150 kHz) concurrent with standard of care treatment for stage 4 non-small cell lung cancer (NSCLC) following platinum failure: the phase III LUNAR study [Abstract CT173]. *Cancer Res*. **79** (2019).

[92] Birch JL, Strathdee K, Gilmour L, Vallatos A, McDonald L, Kouzeli A (2018). A novel small-molecule inhibitor of MRCK prevents radiation-driven invasion in glioblastoma. Cancer Res..

[93] Singh SK, Hawkins C, Clarke ID, Squire JA, Bayani J, Hide T (2004). Identification of human brain tumour initiating cells. Nature.

[94] Bao S, Wu Q, McLendon RE, Hao Y, Shi Q, Hjelmeland AB (2006). Glioma stem cells promote radioresistance by preferential activation of the DNA damage response. Nature.

[95] Prager BC, Bhargava S, Mahadev V, Hubert CG, Rich JN (2020). Glioblastoma stem cells: driving resilience through chaos. Trends Cancer.

[96] Szachowicz-Petelska, B., Figaszewski, Z. & Lewandowski, W. Mechanisms of transport across cell membranes of complexes contained in antitumour drugs. *Int. J. Pharm*. **222**, 169–182 (2001). 10.1016/s0378-5173(01)00713-x 11427347

[97] Chang E, Patel CB, Pohling C, Young C, Song J, Flores TA (2018). Tumor treating fields increases membrane permeability in glioblastoma cells. Cell Death Discov..

[98] Kessler AF, Salvador E, Domröse D, Burek M, Schaeffer C, Tempel Brami C (2019). Blood brain barrier (BBB) integrity is affected by tumor treating fields (TTFields) in vitro and in vivo. Int. J. Radiat. Oncol..

[99] NIH US National Library of Medicine. ClinicalTrials.gov. https://clinicaltrials.gov (2020).

[100] Ceresoli GL, Aerts JG, Dziadziuszko R, Ramlau R, Cedres S, van Meerbeeck JP (2019). Tumour treating fields in combination with pemetrexed and cisplatin or carboplatin as first-line treatment for unresectable malignant pleural mesothelioma (STELLAR): a multicentre, single-arm phase 2 trial. Lancet Oncol..

[101] Vogelzang NJ, Rusthoven JJ, Symanowski J, Denham C, Kaukel E, Ruffie P (2003). Phase III study of pemetrexed in combination with cisplatin versus cisplatin alone in patients with malignant pleural mesothelioma. J. Clin. Oncol..

[102] US Food and Drug Administration. NovoTTF™-100L System -H180002. https://www.fda.gov/medical-devices/recently-approved-devices/novottftm-100l-system-h180002 (2019).

[103] Adamson PC (2015). Improving the outcome for children with cancer: Development of targeted new agents. CA Cancer J. Clin..

[104] Branter J, Estevez-Cebrero M, Grundy R, Basu S, Smith S (2018). Tumor treating fields (TTFields) have antiproliferative effects on high-grade pediatric brain tumor cell lines [Abstract 4637]. Cancer Res..

[105] O’Connell D, Shen V, Loudon W, Bota DA (2017). First report of tumor treating fields use in combination with bevacizumab in a pediatric patient: a case report. CNS Oncol..

[106] Green AL, Mulcahy Levy JM, Vibhakar R, Hemenway M, Madden J, Foreman N (2017). Tumor treating fields in pediatric high-grade glioma. Child. Nerv. Syst..

[107] Ballo MT, Urman N, Lavy-Shahaf G, Grewal J, Bomzon Z, Toms S (2019). Correlation of tumor treating fields dosimetry to survival outcomes in newly diagnosed glioblastoma: a large-scale numerical simulation-based analysis of data from the phase 3 EF-14 randomized trial. Int. J. Radiat. Oncol..

[108] Kinzel A, Ambrogi M, Varshaver M, Kirson ED (2019). Tumor treating fields for glioblastoma treatment: patient satisfaction and compliance with the second-generation Optune® system. Clin. Med. Insights: Oncol..

[109] Bryant HE, Schultz N, Thomas HD, Parker KM, Flower D, Lopez E (2005). Specific killing of BRCA2-deficient tumours with inhibitors of poly(ADP-ribose) polymerase. Nature.

[110] Farmer H, McCabe N, Lord CJ, Tutt AN, Johnson DA, Richardson TB (2005). Targeting the DNA repair defect in BRCA mutant cells as a therapeutic strategy. Nature.

[111] Helleday T (2011). The underlying mechanism for the PARP and BRCA synthetic lethality: clearing up the misunderstandings. Mol. Oncol..

[112] Patil AA, Sayal P, Depondt M-L, Beveridge RD, Roylance A, Kriplani DH (2014). FANCD2 re-expression is associated with glioma grade and chemical inhibition of the Fanconi Anaemia pathway sensitises gliomas to chemotherapeutic agents. Oncotarget.

[113] Rominiyi, O., Myers, K., Gomez-Roman, N., Lad, N., Dar, D., Jellinek, D. et al. RDNA-12. The Fanconi anaemia (FA) pathway and glioblastoma: a new foundation for DNA damage response targeted combinations [Abstract RNDA-12]. *Neuro. Oncol.* **21**, vi209–vi209 (2019).

[114] MacLeod G, Bozek DA, Rajakulendran N, Monteiro V, Ahmadi M, Steinhart Z (2019). Genome-wide CRISPR-Cas9 screens expose genetic vulnerabilities and mechanisms of temozolomide sensitivity in glioblastoma stem cells. Cell Rep..

[115] Li T, Shukla G, Peng C, Lockamy V, Liu H, Shi W (2018). Dosimetric impact of a tumor treating fields device for glioblastoma patients undergoing simultaneous radiation therapy. Front. Oncol..

[116] Straube C, Oechsner M, Kampfer S, Scharl S, Schmidt-Graf F, Wilkens JJ (2018). Dosimetric impact of tumor treating field (TTField) transducer arrays onto treatment plans for glioblastomas—a planning study. Radiat. Oncol..

[117] Grossman R, Limon D, Bokstein F, Harosh CB, Ram Z (2019). Randomized phase II trial of tumor treating fields plus radiation therapy plus temozolamide compared to radiation therapy plus temozolomide in patients with newly diagnosed glioblastoma [Abstract]. Cancer Res..

[118] Mehta, M., Gondi, V., Ahluwalia, M. & Brown, P. Radiosurgery followed by tumour treating fields (TTFields) for brain metastases (1-10) from NSCLC in the phase III METIS trial. *Ann. Oncol*. **30**, v659 (2019).

[119] Pless M, Droege C, Von Moos R, Salzberg M, Betticher D (2013). A phase I/II trial of tumor treating fields (TTFields) therapy in combination with pemetrexed for advanced non-small cell lung cancer. Lung Cancer.

[120] Ceresoli G, Aerts J, Madrzak J, Dziadziuszko R, Ramlau R, Cedres S (2018). STELLAR—final results of a phase 2 trial of TTFields with chemotherapy for first-line treatment of malignant pleural mesothelioma. J. Thorac. Oncol..

[121] Hanna N, Shepherd FA, Fossella FV, Pereira JR, De Marinis F, von Pawel J (2004). Randomized phase III trial of pemetrexed versus docetaxel in patients with non-small-cell lung cancer previously treated with chemotherapy. J. Clin. Oncol..

[122] Rivera F, Benavides M, Gallego J, Guillen-Ponce C, Lopez-Martin J, Kung M (2019). Tumor treating fields in combination with gemcitabine or gemcitabine plus nab-paclitaxel in pancreatic cancer: results of the PANOVA phase 2 study. Pancreatology.

[123] Von Hoff DD, Ervin T, Arena FP, Chiorean EG, Infante J, Moore M (2013). Increased survival in pancreatic cancer with nab-paclitaxel plus gemcitabine. N. Engl. J. Med..

[124] Picozzi, V., Weinberg, U., Giladi, M., Bomzon, Z. & Kirson E. PANOVA-3: a phase 3 study of tumor treating fields with nab-paclitaxel and gemcitabine for front-line treatment of locally advanced pancreatic adenocarcinoma (LAPC) [Abstract P-260]. *Ann. Oncol*. **30**, mdz155-259 (2019).

[125] Vergote I, von Moos R, Manso L, Van Nieuwenhuysen E, Concin N, Sessa C (2018). Tumor treating fields in combination with paclitaxel in recurrent ovarian carcinoma: results of the INNOVATE pilot study. Gynecol. Oncol..

[126] Hanker LC, Loibl S, Burchardi N, Pfisterer J, Meier W, Pujade-Lauraine E (2012). The impact of second to sixth line therapy on survival of relapsed ovarian cancer after primary taxane/platinum-based therapy. Ann. Oncol..

[127] Kirson ED, Giladi M, Bomzon Z, Weinberg U, Farber O (2018). INNOVATE-3: phase 3 randomized, international study of tumor treating fields (200 kHz) concomitant with weekly paclitaxel for the treatment of platinum-resistant ovarian cancer [Abstract]. J. Clin. Oncol..

[128] Grosu A, Gkika E, Brunner TB, Thimme R, Weinberg U (2019). Phase II HEPANOVA trial of tumor treating fields concomitant with sorafenib for advanced hepatocellular carcinoma [Abstract]. J. Clin. Oncol..

