# Correction: Tumour treating fields therapy for glioblastoma: current advances and future directions

**DOI:** 10.1038/s41416-021-01451-5

**Published:** 2021-06-10

**Authors:** Ola Rominiyi, Aurelie Vanderlinden, Susan Jane Clenton, Caroline Bridgewater, Yahia Al-Tamimi, Spencer James Collis

**Affiliations:** 1 Weston Park Cancer Centre, Department of Oncology & Metabolism, The University of Sheffield Medical School, Sheffield, UK; 2 Department of Neurosurgery, Royal Hallamshire Hospital, Sheffield Teaching Hospitals NHS Foundation Trust, Sheffield, UK; 3 Department of Clinical Oncology, Weston Park Hospital, Sheffield Teaching Hospitals NHS Foundation Trust, Sheffield, UK

**Keywords:** Chemotherapy, Physics, CNS cancer, Targeted therapies, Radiotherapy

Correction to: *British Journal of Cancer* 10.1038/s41416-020-01136-5, published online 4 November 2020

The article Tumour treating fields therapy for glioblastoma: current advances and future directions, written by Ola Rominiyi, Aurelie Vanderlinden, Susan Jane Clenton, Caroline Bridgewater, Yahia Al-Tamimi and Spencer James Collis, was originally published electronically on the publisher’s internet portal on 4 November 2020 without open access. With the author(s)’ decision to opt for Open Choice the copyright of the article changed on 24 May 2021 to © The Author(s) 2021 and the article is forthwith distributed under a Creative Commons Attribution 4.0 International License, which permits use, sharing, adaptation, distribution and reproduction in any medium or format, as long as you give appropriate credit to the original author(s) and the source, provide a link to the Creative Commons licence, and indicate if changes were made. The images or other third party material in this article are included in the article’s Creative Commons licence, unless indicated otherwise in a credit line to the material. If material is not included in the article’s Creative Commons licence and your intended use is not permitted by statutory regulation or exceeds the permitted use, you will need to obtain permission directly from the copyright holder. To view a copy of this licence, visit http://creativecommons.org/licenses/by/4.0/.

